# Population-based comparison of cancer survival outcomes in patients with and without psychiatric disorders

**DOI:** 10.1186/s12888-022-04191-9

**Published:** 2022-08-11

**Authors:** Alexander Benny, Mary McLay, Russell C. Callaghan, Alan Bates, Robert Olson

**Affiliations:** 1 Faculty of Medicine, University of British Columbia, Vancouver, Canada; 2 Faculty of Dentistry, University of British Columbia, Vancouver, Canada; 3 Division of Medical Sciences, University of Northern British Columbia, Prince George, Canada; 4 Department of Psychiatry, BC Cancer, University of British Columbia, Vancouver, Canada; 5 Department of Surgery, BC Cancer, University of British Columbia, 1215 Lethbridge Street, Prince George, BC V2M7E9 Canada

**Keywords:** Psychiatric Disorders, Cancer, Survival, Depression, Schizophrenia, Bipolar Disorder

## Abstract

**Background:**

Individuals with psychiatric disorders (PD) have a high prevalence of tobacco use. Patients with PD also potentially receive substandard care in comparison to the general population. Previous research has shown that individuals with PD have a decreased risk of receiving a tobacco related (TR) cancer diagnosis. To further assess this trend, this study assesses the survival of patients with a TR cancer with or without a PD.

**Materials and methods:**

Our study utilized multiple databases, with methods described elsewhere,^6^ to identify people in British Columbia that have been diagnosed with psychiatric disorders and appendicitis (our control group). From these groups, we selected individuals who also had a TR cancer. We subsequently extracted information pertaining to these patients from these databases.

**Results:**

Thirty-nine thousand eight hundred forty-one patients with cancer were included in our study. Analyses of these patients were controlled for by age, gender, cancer type and diagnosis year. This analysis displayed shorter survival time among patients who were diagnosed with depression (HR = 1.16; *p* = 0.01; 95% CI: 1.04–1.29*)*, schizophrenia (HR = 1.62; *p* < 0.01; 95% CI: 1.43–1.84*)*, or bipolar disorder (HR = 1.35; p < 0.01; 95% CI: 1.12–1.64*)* compared to the cancer patients without a PD, all of which were statistically significant. People that were diagnosed with anxiety disorders did not have a survival time that was significantly different from our control population (HR = 1.07; *p* = 0.22; 95% CI: 0.96–1.19).

**Conclusions:**

Individuals with PD, except for those with anxiety, were found to have a shorter survival time following diagnosis with a TR cancer as compared to our control group. We hypothesize several factors, which may account for this statistically significant difference: (1) delayed diagnosis, (2) poor access to care, (3) poor assessment or follow-up, or (4) physician beliefs of poor treatment adherence.

## Key points

### Question

Is there a difference in the survival time of tobacco-related (TR) cancer patients who do or do not have a concurrent psychiatric disorder (PD)?

### Findings

Thirty-nine thousand eight hundred forty-one cancer patients were included in our population-based retrospective cohort study. After controlling for age, gender, cancer type and diagnosis year, there was a significantly shorter survival time among patients diagnosed with depression, schizophrenia, or bipolar disorder compared to the cancer patients without a PD.

### Meaning

We believe that this difference might be due to delayed diagnosis at a later stage, poorer access to care, poorer assessment or follow-up, or physician beliefs of poorer treatment adherence, all of which may contribute to worse survival.

## Introduction

Psychiatric disorders (PD) are a broad class of morbidities related to an individual’s psychological and/or behavioural experience of the world [1]. According to the Mental Health Commission of Canada, 1 in 5 people in Canada currently experience such PD [2], This high prevalence leads to a significant financial strain on our healthcare system [2], not to mention the significant distress and financial toxicity PD cause for the individuals experiencing them, as well as their support systems. In numerous studies, individuals with PD have a higher prevalence of tobacco use compared to the general population [3–5]. One would expect that individuals with PD would, therefore, have a higher risk of developing a tobacco-related (TR) cancer; however, based upon our previous research, this is not the case [6].

We previously reported a study using several population-based databases to compare the risk of developing a TR cancer in those with PD (*n* = 123,752) to a primary population proxy group of those with appendicitis (*n* = 41,537) [6]. We found that individuals with depression (HR = 0.81, *p* < 0.01, 95% CI:0.73–0.91), anxiety disorders (HR = 0.84, *p* = 0.02, 95% CI: 0.73–0.97), or multiple PD diagnoses (HR = 0.74, *p* < 0.01, 95% CI: 0.66–0.83) had a lower risk of developing TR cancers. We hypothesized that the decreased risk was due to barriers those with PD have when accessing care, thus leading to a decreased chance of receiving a cancer diagnosis. This led us to further hypothesize that individuals with PD who do receive a TR cancer diagnosis likely continue to experience barriers to care, resulting in less favourable morbidity and mortality-related outcomes.

This study seeks to compare the survival times of individuals with PD diagnosed with a TR cancer to a proxy control group of individuals with appendicitis diagnosed with a TR cancer. We hypothesize that patients with cancer and a PD will have a significantly shorter survival time from first cancer diagnosis until death than patients with cancer and no PD diagnosis. By studying the survival times of these groups of individuals, we will be able to further describe the understudied relationship between PD, TR cancer, and mortality.

### Major hypotheses

We hypothesize that patients with a TR cancer and PD diagnoses will have a significantly shorter survival time than individuals with a TR cancer and no concurrent PD diagnosis.

## Materials and methods

### Study population: data sources

We conducted a retrospective cohort study using data from several population-based provincial databases, as described elsewhere [6]. All individuals aged 13 to 85 in British Columbia (BC) diagnosed with depression, schizophrenia and related disorders, bipolar disorder, anxiety disorders or multiple PD between January 1, 1990 and December 31, 2013 were selected (refer to Table 1 for ICD codes and cohort criteria). A primary population-proxy control group (appendicitis) was also identified using ICD codes from the Medical Services Plan (MSP) and Discharge Abstracts Data (DAD) databases (Table 2) [7, 8]. To be assigned to the appendicitis cohort, individuals could not have an ICD code for any PD in MSP or DAD databases during the study period.

**Table 1:** Cohort demographic and follow-up information, comparing A) all psychiatric disorders and B) psychiatric disorder sub-cohorts to the appendicitis control group

Characteristic	Control	A)		B)					
	(appendicitis) ***N*** = 664^b^	All PD^c^ ***N*** = 39,177	*P*-value	Depression ***N*** = 15,421	Schizophrenia ***N*** = 840	Bipolar ***N*** = 186	Anxiety ***N*** = 7522	Multiple PD ***N*** = 15,208	*P*-value
Median age at diagnosis in years (range)	67 (15–85)	69 (14–85)	0.02	69 (17–85)	74 (17–85)	69 (29–85)	70 (19–85)	67 (14–85)	< 0.001
Male, *n* (%)	437 (66%)	17,336 (44%)	< 0.001	7050 (46%)	493 (59%)	87 (47%)	3747 (50%)	5959 (39%)	< 0.001
Cancer type, *n* (%)									
Lung	125 (19%)	13,786 (35%)	< 0.001	5599 (36%)	337 (40%)	79 (43%)	2680 (36%)	5091 (34%)	< 0.001
Colorectal	365 (55%)	10,988 (28%)		4138 (27%)	208 (25%)	45 (24%)	2060 (27%)	4537 (30%)	
Esophagus	14 (2%)	869 (2%)		354 (2%)	26 (3%)	a	182 (2%)	304 (2%)	
Stomach	23 (4%)	1677 (4%)		679 (4%)	60 (7%)	7 (4%)	348 (5%)	583 (4%)	
Lip, oral cavity, pharynx	18 (3%)	1968 (5%)		715 (5%)	25 (3%)	9 (5%)	353 (5%)	866 (6%)	
Liver	19 (3%)	952 (2%)		404 (3%)	31 (4%)	a	174 (2%)	340 (2%)	
Pancreas	29 (4%)	2520 (6%)		1087 (7%)	47 (6%)	12 (7%)	523 (7%)	851 (6%)	
Larynx	7 (1%)	448 (1%)		196 (1%)	7 (1%)	a	79 (1%)	164 (1%)	
Cervix	11 (2%)	1006 (3%)		321 (2%)	10 (1%)	a	141 (2%)	531 (4%)	
Kidney	27 (4%)	2308 (6%)		846 (6%)	28 (3%)	9 (5%)	437 (6%)	988 (7%)	
Bladder	26 (4%)	2101 (5%)		859 (6%)	53 (6%)	14 (8%)	425 (6%)	750 (5%)	
AML	a	554 (1%)		223 (1%)	8 (1%)	a	120 (2%)	203 (1%)	
Mean years of follow-up (to death)	6.7	8.8	< 0.001	8.9	9.1	9.5	8.8	8.6	< 0.001
Died during study period, *n* (%)	359 (54%)	25,230 (64%)	< 0.001	10,425 (68%)	710 (85%)	148 (80%)	5045 (67%)	8902 (59%)	< 0.001
Tobacco-related deaths, *n* (%)^d^									
Total person-years of follow-up	2601	123,308	–	42,406	1440	579	22,105	56,779	–

All PD = any psychiatric disorder diagnosis; Schizophrenia = schizophrenia and related diseases; Bipolar = bipolar disorder; Anxiety = anxiety disorders; Multiple PD = diagnosis with 2 or more PD on same day

^a^cell sizes less than 5 are suppressed as per Population Data BC guidelines

^b^Appendicitis cases were identified using ICD-9 codes: 540–542 and ICD-10 codes: K35-K37

^c^PD cases were identified using ICD-9 codes: Psychotic Conditions: (293.84295296.0–296.8297298), Neurotic Disorders (300.0–300.5300.89300.9), Dependence Syndromes (303,304.0, 304.2, 304.3, 304.4), Non-dependent Substance Abuse (305.0, 305.2, 305.5, 305.6, 305.7), Acute Reactions to Stress and Adjustment Reaction (308.0–308.9309.8), Depressive Disorder (311), Disturbance of Emotions Related to Childhood and Adolescence (313.0–313.83), Poisoning by Substances (965.0968.5969.7980.0), Other (E850.0, E850.1, E850.2 E854.2 E855.2) and ICD-10 codes: Organic Mental Disorders (F06.4), Mental and Behavioural Disorders due to Psychoactive Substance Use (F10 F11.1, F11.2 F12.1, F12.2 F14.1, F14.2 F15.1, F15.2), Schizophrenia, Schizotypal, and Delusional Disorders (F20 F21 F22 F24 F28 F29), Mood Disorders (F30 F31.0, F31.1, F31.2, F31.3, F31.4, F31.5, F31.6, F31.8, F31.9 F32.2, F32.3, F32.8 F33.2, F33.3, F33.4, F33.8, F33.9 F34.1 F38.1), Neurotic, Stress-Related and Somatoform Disoders (F40 F41 F42 F43.0, F43.1, F43.9 F44 F48.0, F48.8, F48.9), Postnatal and Postpartum Depression (F53.0), Disturbance of Emotions and Behaviour Related to Childhood and Adolescence (F93, F94), Poisoning (T40.0, T40.1, T40.3, T40.4, T40.5 T43.6 T51.0 X41 X42)

^d^Tobacco-related deaths were identified using the ICD 9 codes: Malignant Neoplasms of Lip, Oral Cavity and Pharynx (140–149), Malignant Neoplasm of Digestive Organs and Peritoneum (150, 151, 157), Malignant Neoplasm of Respiratory and Intrathoracic Organs (161, 162), Malignant Neoplasm of Genitourinary Organs (180, 188, 189), Malignant Neoplasm of Lymphatic and Haematopoietic Tissue (205.0), Ischemic Heart Disease (410–414), Cardiovascular Disease (unspecified; 429.2), Acute Rheumatic Fever and Chronic Rheumatic Heart Disease (390–398), Disease of Pulmonary Circulation (415–417), Other Forms of Heart Disease (420–429.1, 429.3–429.9), Cerebrovascular Disease (430–438), Disease of Arteries, Arterioles, and Capillaries (440, 441, 442–448), Pneumonia and Influenza (480–487), Chronic Obstructive Pulmonary Disease and Allied Conditions (490–492, 496) and ICD-10 codes: Malignant Neoplasm of Lip, Oral Cavity, and Pharynx (C00-C14), Malignant Neoplasm of Digestive Organs (C15, C16, C25), Malignant Neoplasm of Respiratory and Intrathoracic Organs (C32-C34), Malignant Neoplasm of Female Genital Organs (C53), Malignant Neoplasm of Urinary Tract (C64, C65, C67), Malignant Neoplasm of Lymphoid, Haematopoietic, and Related Tissue (C92.0), Influenza and Pneumonia (J10-J18), Chronic Lower Respiratory Diseases (J40-J44), Other (120–125, 100–109, 126–151, 160–169, 170–178)

**Table 2:** Multivariable analysis to assess the effect of PD diagnosis on overall and TR cancer-specific survival, controlling for age, gender, cancer type and diagnosis year

		Tobacco-Related Cancer-Specific Survival			Overall Survival		
		Hazard Ratio	95% Confidence Interval	***P***-Value	Hazard Ratio	95% Confidence Interval	***P***-Value
Psychiatric disorder	Appendicitis (control)	1	reference	–	1	reference	–
	Depression	1.13	0.99–1.29	0.07	1.16	1.04–1.29	< 0.01
	Schizophrenia	1.56	1.33–1.83	< 0.01	1.62	1.43–1.84	< 0.01
	Bipolar disorder	1.20	0.92–1.57	0.19	1.35	1.12–1.64	< 0.01
	Anxiety	1.07	0.94–1.23	0.29	1.07	0.96–1.19	0.24
Age (continuous yearly)		1.02	(1.02–1.02)	< 0.01	1.03	1.03–1.03	< 0.01
Gender	Male	1	reference	–	1	reference	–
	Female	0.93	(0.90–0.97)	< 0.01	0.90	0.88–0.93	< 0.01
Diagnosis year		1.06	1.05–1.06	< 0.01	0.98	0.98–0.98	< 0.01
Cancer type	Lung	1	reference	–	1	reference	–
	Lip, oral cavity, pharynx	0.26	0.23–0.29	< 0.01	0.32	0.29–0.35	< 0.01
	Esophagus	0.92	0.82–1.02	0.11	0.92	0.84–1.01	0.09
	Stomach	0.74	0.68–0.81	< 0.01	0.75	0.70–0.81	< 0.01
	Colorectal	0.28	0.27–0.30	< 0.01	0.32	0.31–0.34	< 0.01
	Liver	0.96	0.86–1.06	0.41	1.12	1.02–1.22	0.02
	Pancreas	1.65	1.55–1.76	< 0.01	1.64	1.55–1.73	< 0.01
	Larynx	0.24	0.19–0.29	< 0.01	0.28	0.23–0.32	< 0.01
	Cervix	0.22	0.18–0.27	< 0.01	0.27	0.23–0.32	< 0.01
	Kidney	0.26	0.23–0.29	< 0.01	0.33	0.30–0.36	< 0.01
	Bladder	0.29	0.26–0.32	< 0.01	0.36	0.34–0.39	< 0.01
	AML	1.08	0.94–1.25	0.29	1.30	1.16–1.46	< 0.01

Schizophrenia = schizophrenia and related diseases; Anxiety = anxiety disorders

Individuals with appendicitis were used as a primary population-proxy control group for our study, as according to previous research [9]. Individuals with appendicitis were chosen as our control group for a multitude of reasons. Appendicitis is a common reason for admission to hospital; therefore, it provides a large enough sample for us to match to our target cohort (those with PD). It is important that we use inpatient appendicitis data, as past research has displayed that using control groups outside of hospital can bias hazard ratio estimations [10]. Additionally, appendicitis does not appear to be related to socioeconomic status [11]. Finally, previous epidemiological studies have included individuals with appendicitis as a control group successfully [12–15].

**Fig. 1:**
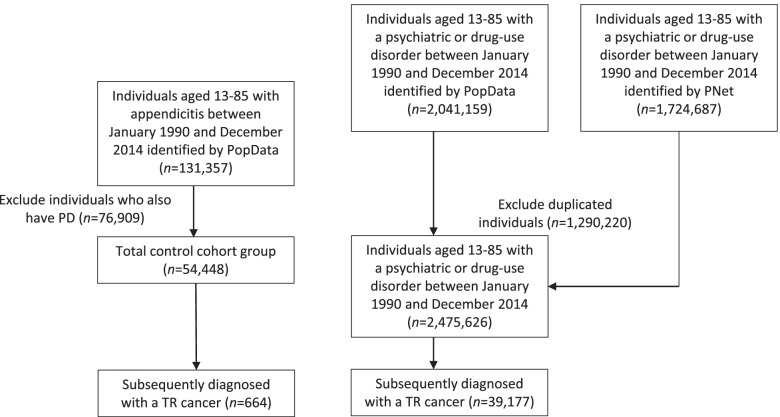
Selection process for patients included in our study

We then followed all individuals with either a PD or appendicitis diagnosis for subsequent development of a TR cancer by linking with the BC Cancer Registry (BCCR) database [16], which captures all incident cancer cases in the province. The following twelve cancers shown to be causally linked to smoking were selected: oropharyngeal, laryngeal, esophageal, lung and bronchial, acute myeloid leukemia (AML), stomach, liver, pancreatic, kidney and renal pelvis, cervix, urinary bladder and colorectal. All individuals diagnosed with a TR cancer (and prior PD or appendicitis diagnosis) were used as the cohort for this study (Fig. 1).

### Measurement of outcomes

The PD and control cancer cohorts were linked with the Vital Statistics Deaths database, where all deaths of BC residents are reported.

### Statistical methods

Descriptive statistics were performed. Overall survival (OS) rates were estimated by the Kaplan-Meier method and survival curves. Survival was compared between the control group and PD cohorts using the log-rank test. Multivariable survival analyses were performed using Cox regression models to assess PD and appendicitis group differences in survival, while controlling for age, gender, cancer type, and diagnosis year. For survival analyses, according to previous methodologies [6], time was calculated from the date of TR cancer diagnosis until death. If subjects were still alive at the end of the study period, the censor time was calculated as the time from diagnosis until December 31, 2014, since all deaths in Canada are accurate up to this date at the time of the analysis.

The statistical tests were two-sided. *P*-values that were less than or equal to 0.05 were considered significant in our study. The Statistical Package for Social Sciences software version 23.0 (SPSS, Chicago, IL) was used to conduct our statistical analyses. Approval for this study was provided by the joint Research Ethics Boards of the University of British Columbia and BC Cancer Agency.

## Results

### Clinical characteristics

Thirty-nine thousand one hundred seventy-seven patients diagnosed with a PD between 1990 and 2014 in BC were subsequently diagnosed with a TR cancer. During the same time, 664 patients diagnosed with appendicitis were diagnosed with a TR cancer. In both the PD and appendicitis cohorts, the majority of cancer diagnoses were either lung (35 and 19%, respectively) or colorectal cancer (28 and 55%, respectively). Table 1 shows further patient characteristics and follow-up information for these two cohorts. Figure 1 displays a graphic on how patients were included in our study.

### Survival analysis

**Fig. 2:**
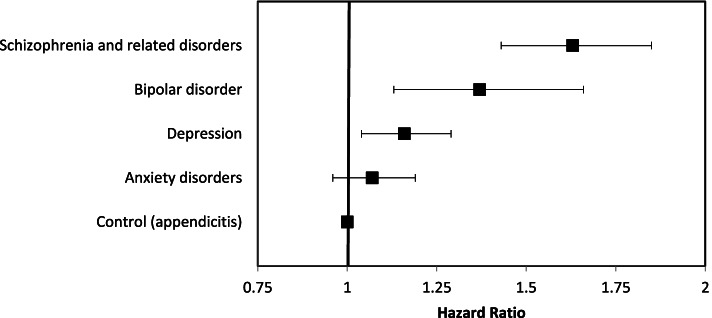
Impact of psychiatric disorder on overall survival, controlling for age, gender, cancer type and diagnosis year. Visual representation of hazard ratio and 95% confidence interval from Cox proportion hazard model

**Fig. 3:**
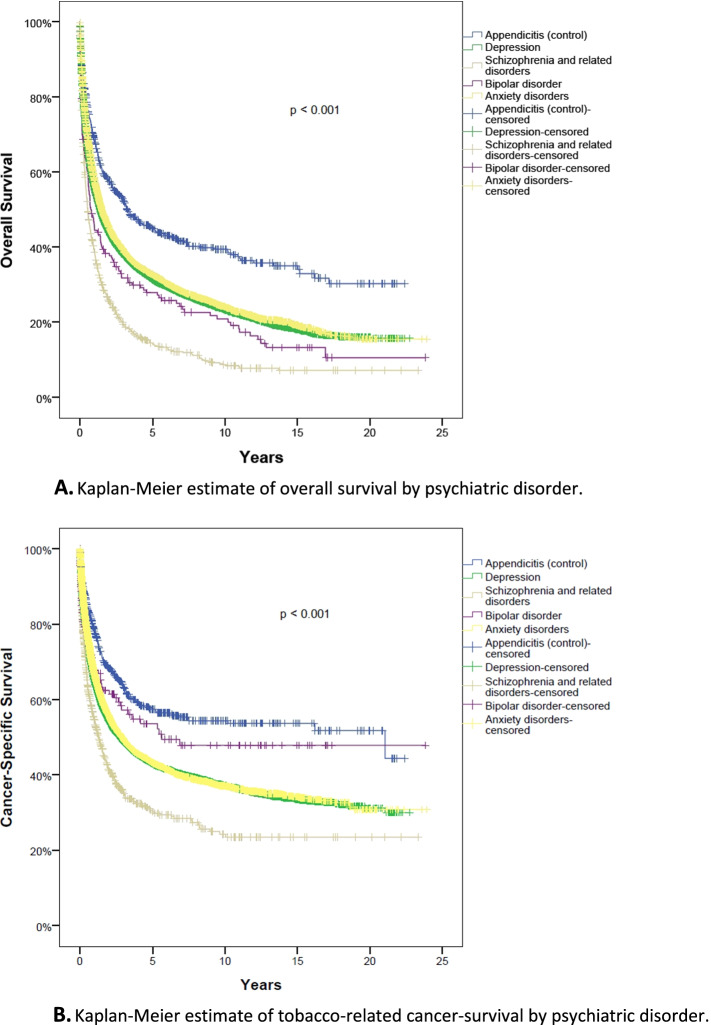
**A** Kaplan-Meier estimate of overall survival by psychiatric disorder. **B** Kaplan-Meier estimate of tobacco-related cancer-survival by psychiatric disorder

The 5-year OS for the depression, schizophrenia and related disorders, bipolar disorder, anxiety disorders, and multiple PD sub-cohorts were 31, 14, 28, 45 and 44%, respectively. The appendicitis cohort had a 5-year OS of 45%. There was a significant difference in OS between the control and PD cohorts (*p* < 0.001; Figs. 2 and 3).

On multivariable analysis, after controlling for age, gender, cancer type and diagnosis year, OS was significantly worse among patients in the depression, schizophrenia and related disorders, and bipolar disorder cohorts compared to the control group (HR range = 1.07–1.63; p < 0.001; Table 2). OS among individuals with anxiety disorders, however, did not significantly differ from the control group (HR = 1.07; 95% CI: 0.96–1.19; *p* = 0.25).

## Discussion

In this large, population-based study we found that overall survival (OS) was significantly lower for patients with cancer and comorbid depression, schizophrenia and related disorders, or bipolar disorder diagnoses compared to the patients with cancer from a population-proxy control group without PD. We theorize that the decreased OS for individuals with PD may be due to delayed diagnosis at a later stage, poorer access to care, poorer assessment, or physician beliefs of poorer capacity for treatment adherence, all of which may contribute to worse survival.

Our findings of increased mortality in individuals with concurrent PD aligns with previous work. Specific to TR mortality, Callaghan et al. [17] conducted a large cohort study that used standardized mortality ratios to compare TR mortality for inpatients diagnosed with schizophrenia, bipolar disorder, or major depressive disorder to the general population. Similar to our findings, the standardized mortality ratios related to TR conditions for all of the PD cohorts exceeded that of the standard population (schizophrenia = 2.45, 95% CI: 2.41–2.48; bipolar disorder = 1.57, 95% CI: 1.53–1.62; major depressive disorder = 1.95, 95% CI: 1.93–1.98). Our results suggest this phenomenon applies not only to PD patients ill enough to require inpatient psychiatric care, but to a wider spectrum of PD severity, even within a Canadian cancer control system where financial barriers to care may be less prominent.

Additionally, a population-based cohort study out of France studied whether end-of-life care offered to those with schizophrenia and cancer differed from those with cancer and no concurrent PD, as well as survival outcomes related to these groups [18]. Individuals with schizophrenia and cancer were matched to controls (cancer patients without schizophrenia) based upon age of death, sex, and primary cancer location. It was found that individuals with schizophrenia had a significantly reduced median time from cancer diagnosis to death (schizophrenia = 290 days, IQR: 70–714; control = 327 days IQR: 70–780). Additionally, in the unmatched analyses, patients with cancer and schizophrenia died at a younger mean age than those with cancer and no schizophrenia (schizophrenia = 63.6 years, SD: 12.8; control = 71.8 years, SD: 12.7). These findings, in concordance with our own results, portray that there are mortality disparities between those with cancer and PD and those with cancer and no PD. This work by Fond et al. suggest that this trend is not only applicable to TR cancers, but perhaps cancer in general, suggesting that this issue is more widespread and multi-faceted.

A review by Knaak et al. speaks to the stigma individuals with mental illness face when accessing healthcare [19]. They note that individuals with PD “report feeling devalued, dismissed, and dehumanized by many health professionals” [19], which undoubtedly causes barriers to care. In some cases, healthcare providers neglect whole-person care in PD, instead tending to view presentations of various medical syndromes as by-products of their psychiatric illness [19]. This “illness-first” approach leads to healthcare professionals not taking appropriate caution when assessing non-mental health issues (a process termed diagnostic overshadowing) [20]. Another review has commented on similar trends, specifically in a study where American family physicians were less likely to believe physical symptom reports of patients with a history of depression, ultimately leading to the omission of appropriate investigations [20]. We posit that, though there are wider societal factors such as social isolation that disadvantage overall support for people with PD, this stigma within healthcare towards those with PD also contributes to decreased overall survival through substandard assessment, delayed diagnosis, and poor access to specialized care. Our results can help support this claim. Table 1 shows that a higher proportion patients with PD had lung cancer (35% compared to 19% in our control group) rather than colorectal cancer (28% compared to 55% in our control group). Perhaps this decreased proportion of colorectal cancer in those with PD speaks to less uptake in provincial colorectal screening programs, future researchers could explore this avenue.

PD-related stigma is likely compounded by stigma faced by people who smoke. Extensive anti-smoking policies and negative social perceptions, while good intentioned, disproportionately affect those who are already stigmatized [21]. This intersectionality of social burden could possibly further reduce access to receiving quality care. Unfortunately, anti-smoking measures appear to have reduced impact for individuals with PD, as shown by the slow decline in smoking rates for these individuals [4, 5], while contributing to the stigma they face [21].

Stigma not only extends to accessing care (i.e., getting a diagnosis), but also to receiving care. A systematic review by Henderson et al. remarked on a study where primary care providers’ attitudes led to inappropriate prescribing practices for patients with schizophrenia, based upon belief those patients would be unable to adhere to a treatment regime [20]. It’s likely PD patients included in our analysis were affected by similar beliefs. Foti and colleagues have demonstrated effective healthcare decision making in even severe and persistent mental illness, but it’s unfortunately common for PD patients to not be offered more aggressive treatment options due to concerns about capacity for informed decision-making [22]. Some healthcare workers even confess they avoid attending to patients with PD, due to a perception these patients are more violent than other patients [20], despite evidence people with PD are much more likely to be victims of violence than perpetrators.

Importantly, individuals with anxiety did not have significantly different OS than our control population. We theorize that this is due to the nature of anxiety disorders. A large proportion of individuals with anxiety are identified as “help-seeking”, and therefore may be better able to advocate for themselves than patients with other PD. Futhermore, with better-than average follow-up, these individuals may be perceived by practitioners as more suitable for interventions that could increase survival, compared to patients with other PD. Future research should be employed to further describe why individuals with anxiety did not have significantly different OS than those without PD.

This study should be assessed in the context of its strengths and limitations. While there is reason to believe delayed diagnosis at a later stage, poorer access to care, poorer assessment or follow-up, and/or physician beliefs of inability to understand or adhere to treatment options may contribute to our findings – our study did not gather data to confirm such hypotheses. We were also not able to obtain detailed smoking history for the individuals included in this study. Therefore, we were not able to confirm if our cohorts of individuals with PD had higher premorbid tobacco use than our control group, or were less likely to quit after cancer diagnosis, both of which could have contributed to our outcomes. Additionally, our dataset relies on death registration by physicians. As we studied a marginalized and discriminated population, there is reason to believe that there could be difference in the quality of registration in those with PD as compared to our control population. However, our dataset is population-based and includes information on all of BC’s residents. We recommend future research consider such variables in study design. However, we believe this study utilizing a large, high-quality, population-based database within a Canadian healthcare system expands generalizability of some findings compared to previous work. Our findings add to the growing evidence that PD, particularly when combined with tobacco use disorder, should be a focus of efforts to provide equitable cancer care resulting in both improved quality of life and survival for patients with mental health and addictions comorbidities.

## Conclusions

Patients with depression, schizophrenia or bipolar disorder diagnoses had significantly shorter survival time upon diagnosis with TR cancer. We believe this difference might be due to delayed diagnosis at a later stage, poorer access to care, poorer assessment or follow-up, and/or physician beliefs about patient capacity to understand or adhere to treatment, all of which may contribute to worse survival. These results build on the understudied relationships between PD, tobacco use and cancer mortality. Future researchers could explore individual cancer types to study the variation in the relationship between specific TR cancers and individuals with PD. We recommend that further research attempt to further evaluate the possible causal relationships between PD-related factors and worse outcomes, thereby leading to specific interventions to improve outcomes for patients with PD and cancer.

## Data Availability

Access to data provided by the Data Steward(s) at PopulationData British Columbia is subject to approval, but can be requested for research projects through the Data Steward(s) or their designated service providers. The data stewards can be contacted at dataaccess@popdata.bc.ca. All inferences, opinions, and conclusions drawn in this publication are those of the author(s), and do not reflect the opinions or policies of the Data Steward(s).
